# Physical mapping of 5S and 18S ribosomal DNA in three species of *Agave* (Asparagales, Asparagaceae)

**DOI:** 10.3897/CompCytogen.v7i3.5337

**Published:** 2013-08-12

**Authors:** Victor Manuel Gomez-Rodriguez, Benjamin Rodriguez-Garay, Guadalupe Palomino, Javier Martínez, Rodrigo Barba-Gonzalez

**Affiliations:** 1 Centro de Investigación y Asistencia en Tecnología y Diseño del Estado de Jalisco A.C., Unidad de Biotecnología Vegetal. Av. Normalistas No. 800. C.P. 44270. Guadalajara, Jalisco. Mexico; 2 Instituto de Biología, Jardín Botánico, Universidad Nacional Autónoma de México, México D. F., C.P. 04510, Mexico

**Keywords:** Agave, Fluorescent *In Situ* Hybridization, Ribosomal DNA, Karyotype, Physical mapping

## Abstract

*Agave* Linnaeus, 1753 is endemic of America and is considered one of the most important crops in Mexico due to its key role in the country’s economy. Cytogenetic analysis was carried out in *Agave tequilana* Weber, 1902 ‘Azul’, *Agave cupreata* Trelease et Berger, 1915 and *Agave angustifolia* Haworth, 1812. The analysis showed that in all species the diploid chromosome number was 2n = 60, with bimodal karyotypes composed of five pairs of large chromosomes and 25 pairs of small chromosomes. Furthermore, different karyotypical formulae as well as a secondary constriction in a large chromosome pair were found in all species. Fluorescent *in situ* hybridization (FISH) was used for physical mapping of 5S and 18S ribosomal DNA (rDNA). All species analyzed showed that 5S rDNA was located in both arms of a small chromosome pair, while 18S rDNA was associated with the secondary constriction of a large chromosome pair. Data of FISH analysis provides new information about the position and number of rDNA *loci* and helps for detection of hybrids in breeding programs as well as evolutionary studies.

## Introduction

*Agave* Linnaeus, 1753 is a genus of the monocotyledonous family Asparagaceae, belonging to the subfamily Agavoideae (APGIII 2009). It is distributed from southern U.S.A. to Colombia and Venezuela, including the Caribbean Islands (García-Mendoza 2002). The genus has a basic chromosome number x = 30 (Doughty 1936, Brandham 1969, Ruvalcaba-Ruiz and Rodriguez-Garay 2002) and diploid to hexaploid species have been reported (Banerjee and Sharma 1987, Castorena-Sánchez et al. 1991, Palomino et al. 2005, Palomino et al. 2012). Species of this genus are characterized by asymmetric and highly conserved bimodal karyotypes, which consist in five pairs of large chromosomes and 25 pairs of small chromosomes, maintaining the same karyotype structure (Castorena-Sánchez et al. 1991, Brandham and Doherty 1998, Moreno-Salazar et al. 2007, Palomino et al. 2010).

Fluorescent *in situ* hybridization (FISH) is a very useful technique in plant cytogenetics for the physical mapping of multigene families (Mukai et al. 1991) and DNA sequences to plant chromosomes (Rayburn and Gill 1985) as well as chromosome identification (Brown et al. 1999, Hizume et al. 2002, Koo et al. 2004, Kato et al. 2004). The ribosomal RNA (rRNA) genes have been used as probes in FISH because of the high copy number of repeat units, specific position in chromosomes and highly conserved sequences (Liu and Davis 2011). Plant rDNA consists of the 18S, 5.8S and 26S (45S) and 5S genes; in yeasts, these genes are juxtaposed in the same *locus*, whereas in higher eukaryotes, they are organized as families of tandemly repeated units located at one or a few chromosomal sites (Lavania et al. 2005, Garcia et al. 2009). 45S rRNA genes are clustered in tandem arrays of repeat units of 18S, 5.8S and 26S genes, internal transcribed spacers (ITS) and external non-transcribed spacers (NTS), with an approximate size of 7.5–18.5 Kb in plants (Mizuochi et al. 2007). 5S rRNA genes also occur in high numbers as tandem repeats, usually independent of 45S rDNA, however, co-localization of 45S and 5S rDNA have been reported in some angiosperms as *Silene chalcedonica* E.H.L. Krause, 1901 (Siroky et al. 2001) and *Artemisia* Linnaeus, 1753 (Garcia et al. 2007); 5S rDNA repeat unit size ranges between 0.2-0.9 Kb, with a highly conserved region (120 bp in length) separated by a NTS (Specht et al. 1997). These genes are highly conserved, so they have been used as molecular markers in a large number of plant species, such as *Triticum* Linnaeus, 1753 (Jiang and Gill 1994), *Gossypium hirsutum* Linnaeus, 1763 (Ji et al. 1999), *Hordeum vulgare* Linnaeus, 1753 ‘Plaisant’ (Cuadrado and Jouve 2010); however, comparative studies using rDNA as markers in *Agave* have been limited, such as those by Robert et al. (2008), where they reported the number of rDNA *loci* in a few species and demonstrated the existence of additivity in the number of *loci* with increasing ploidy.

The aim of this work was to identify the number and chromosomal location of rDNA sites in three different species of the genus *Agave* including *Agave tequilana* Weber, 1902 ‘Azul’, *Agave angustifolia* Haworth, 1812 ‘Lineño’ and ‘Cimarron’ and *Agave cupreata* Trelease et Berger, 1915 by physical mapping of 5S and 18S rDNA from *Agave tequilana* ‘Azul’.

## Methods

### Plant material

Plants were collected in the Denomination of Origin Zone for *Agave tequilana* ‘Azul’ and in southern Jalisco, México (municipality of Tolimán) for *Agave angustifolia* ‘Lineño’ and ‘Cimarron’ and in Miraval, Guerrero for *Agave cupreata*. Three accessions of each species and varieties were used in this work; the accessions were planted in pots containing a mixture of organic soil:sand:vermiculite (3:3:1) and kept under standard greenhouse conditions.

### Mitotic chromosome counts

Elongating secondary root tips were treated with 2 mM 8-hydroxyquinoleine for 6 hours at 18 °C, in darkness. Later, root tips were fixed in ethanol:acetic acid (3:1) for 24 hours. Root tips were hydrolyzed with 1 N HCl for 15 minutes at 60 °C, transferred to Schiff’s reagent for 1 hour, and then to 1.8% propionic orcein to stain chromosomes (Moreno-Salazar et al. 2007). Slides were frozen with dry ice (Conger and Fairchild 1953), and mounted in Canada balsam. Twelve of the best cells of each population were photographed by using Technical Pan Film and a Zeiss photomicroscope II (Carl Zeiss AG, Germany).

### Karyotype analysis

A negative film was used to draw and measure the chromosome arms and the total genome length. The centromere position was obtained following Levan et al. (1964); arm ratio (r = long arm/short arm) was calculated for each chromosome. Chromosome homology was assigned according to similarities in length and centromere position. In addition, secondary constrictions were useful to distinguish homologous pairs in all populations. Idiograms were constructed according to the arm ratio of the chromosomes, and then grouped in metacentric (m), submetacentric (sm), subtelocentric (st) and telocentric (t) chromosomes. The number of homologous chromosomes was sequentially assigned following chromosome length, for a total number of 30.

### Chromosome preparations

Root tips of each three accessions of *Agave tequilana* ‘Azul’, *Agave angustifolia* ‘Lineño’ and *Agave angustifolia* ‘Cimarron’ and *Agave cupreata* were collected early in the morning, pretreated with satured α-bromonaphthalene solution and kept in ice water overnight, then fixed in ethanol:acetic acid (3:1), for at least 12 hours and stored at -20 °C until use. Root tips were incubated in a pectolytic-enzyme mixture, containing 0.2% (*w*/*v*) pectolyase (Sigma, USA), 0.2% (*w*/*v*) cellulase Onozuka RS (Yakult, Japan), and 0.2% (*w*/*v*) cytohelicase (Sigma) in 10 mM citrate buffer (pH 4.5), at 37 °C for approximately 2 hours. Squash preparations were made in a drop of 45% acetic acid and frozen in liquid nitrogen; the cover slips were removed with a razor blade and slides were dehydrated in absolute ethanol and then air-dried. The best slides were stored at 2–3 °C for up to 1 month.

### Amplification and cloning of rDNA from *Agave tequilana* ‘Azul’

Total genomic DNA from *Agave tequilana* ‘Azul’ was extracted from fresh young leaves using the CTAB method (Murray and Thompson 1980). The 5S and 18S rRNA genes were amplified by PCR using the following set of primers as follows: 5SF (5’-CACCAGATCCCATCAGAACT-3’); 5SR (5’-TTAGTCTGGTATGATCGCAC-3’); 18SF (5’-CAAAGATTAAGCCATGCATG-3’) and 18SR (5’-CCCAGAACATCTAAGGGCAT-3’) (Integrated DNA Technologies, USA). Both PCR reactions were performed in 20 µl reactions containing: 5.2 µl mQ water, 2µl *Taq* buffer 10×, 1 µl 50 mM MgCl_2_, 1.6 µl 2.5 mM dNTPs, 2 U *Taq* polimerase (Life Technologies Corporation, USA), 2.5µl 1 mM of each primer and 50 ng DNA (5µl). Cycling conditions for 5S rDNA were: 94 °C for 4 minutes; 35 cycles of 94 °C for 30 s, 55 °C annealing temperature for 30 s and 72 °C for 30 s, followed by a final extension of 72 °C for 10 minutes. Cycling conditions for 18S rDNA were: 94 °C for 5 minutes; 35 cycles of 94 °C for 30 s, 60 °C annealing temperature for 30 s and 72 °C for 90 s, followed by a final extension of 72 °C for 10 minutes. PCR products were separated by 1% agarose gel electrophoresis in 1× TAE running buffer. Products were visualized by staining with ethidium bromide and the most prominent bands (~1400 bp for 18S and 300-500 bp for 5S) were purified by QIAquick Gel Extraction kit (Qiagen, Germany) according to the manufacturer’s instructions. The purified bands were cloned into pGem^®^-T Easy Vector System I (Promega, USA), incubated overnight at 4 °C. Ligation products were transformed into electrocompetent *Escherichia coli* DH5α cells (Life Technologies Corporation). The recombinant clones were sequenced by LANGEBIO (Cinvestav, Irapuato, Mexico). The sequences were edited with BioEdit version 7.0.9 (Ibis Biosciences, USA) and compared with other sequences available in GenBank (http://www.ncbi.nlm.nih.gov/).

### Probe labeling

5S and 18S rDNA probes were isolated with the High Pure Plasmid Isolation kit (Roche Diagnostics GmbH, Germany) and labeled with biotin-16-dUTP by nick translation according to the manufacturer’s instructions (Roche Diagnostics GmbH).

### Fluorescent *in situ* hybridization

*Slide pretreatment*. Slides were incubated in RNase A (100 μg ml^-1^ in 2× SSC) for 1 hour at 37 °C, and washed with 2× SSC for 15 minutes. Then, the slides were incubated in 0.01 M HCl for two minutes and followed by treatment in pepsin (5 μg ml^-1^) in 0.01M HCl for 10 minutes at 37 °C. Afterwards, the slides were washed in 2× SSC for 10 minutes and incubated in 4% paraformaldehyde for 10 minutes at room temperature. Finally, the slides were dehydrated in ethanol series (70%, 90%, and absolute ethanol for 3 minutes each), and air-dried.

*Probe hybridization*. Hybridization was carried by using a mixture consisting of 20× SSC, formamide, 50% sodium dextran sulphate, 10% sodium dodecyl sulphate, and 25-50 ng/slide of each probe. DNA probes were denatured by heating the hybridization mixture at 70 °C for 10 minutes and then placing it on ice for at least 10 minutes. For each slide, 40 μl of the hybridization mixture were used. Slides were denatured at 80 °C for 5 minutes. The slides were then placed in a pre-warmed humid chamber and incubated overnight at 37 °C. Slides were washed at 37 °C in 2× SSC for 15 minutes, 0.1× SSC at 42 °C for 30 minutes, and 2× SSC at room temperature for 10 minutes.

*Signal detection*. Biotin-labeled probes were detected with streptavidin-Alexa Fluor^546^ conjugate (Life Technologies Corporation) and amplified with biotinylated goat-antistreptavidin (Vector Laboratories, USA). Chromosomes were counterstained with DAPI solution (1 μg ml^-1^), and one drop of Vectashield antifade (Vector Laboratories) was added before examination under a Leica DMRA2 microscope (Leica Microsystems, Germany) equipped with epifluorescent illumination and coupled to an Evolution QEi Camera (Media-Cybernetics, USA), and the images were analyzed with the Image-Pro software (Media-Cybernetics) and enhanced with Photoshop (Adobe Systems Incorporated, USA).

## Results

### *Agave tequilana* ‘Azul’ rDNA cloned sequences

The partial amplification of 18S rDNA generated one band, which was cloned into electrocompetent *Escherichia coli* DH5α cells and a single clone was isolated, which after sequencing showed a fragment of 1424 bp (GenBank: KF159807) and a maximal identity of 100 % with *Agave tequilana* cultivar Azul (GenBank: GU980213.1) and *Agave ghiesbreghtii* K.Koch, 1862 voucher Chase 3467(K) (GenBank: HM640709.1) according to BLASTn analysis (nucleotide blast) at the NCBI database. The partial amplification of 5S rDNA generated one band, which was cloned into electrocompetent *Escherichia coli* DH5α cells and one clone was isolated, which after sequencing showed a fragment of 436 bp (GenBank: KF159808) and a maximal identity of 97% with *Arabidopsis thaliana* (Linnaeus, 1753) clone CIC YAC 9A12 and 9A5 5S ribosomal RNA gene (GenBank: AF198223.1), according to BLASTn analysis (nucleotide blast) at the NCBI database.

### *In situ* hybridization

**Figure 1.**
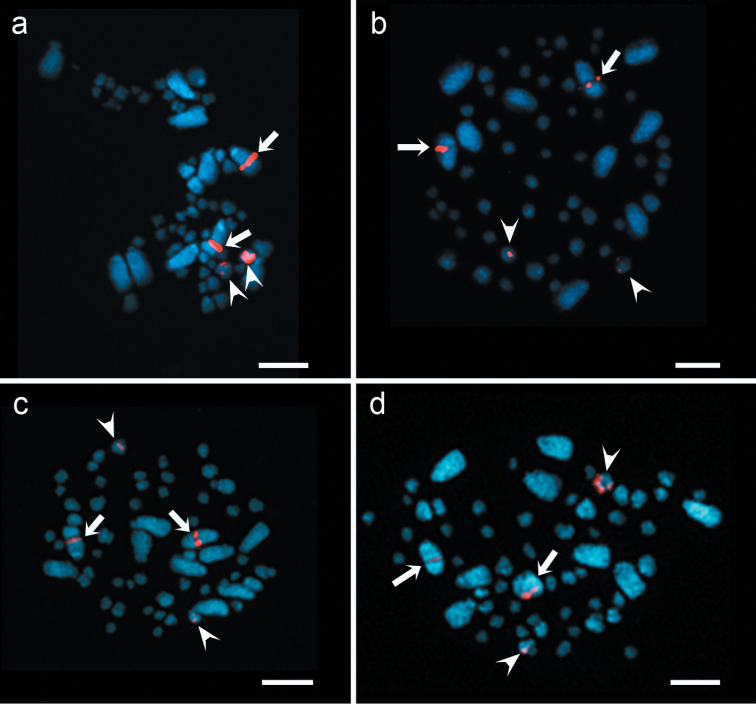
FISH of 5S and 18S rDNA in *Agave* species. Two hybridization sites of 18S rDNA (arrows) and 5S rDNA (arrowheads) in: **a** *Agave tequilana* ‘Azul’ **b** *Agave cupreata* **c** *Agave angustifolia* ‘Lineño’ **d** *Agave angustifolia* ‘Cimarron’. Bars = 10 µm.

The physical mapping of 5S and 18S rDNA from *Agave tequilana* ‘Azul’ were investigated by fluorescent *in situ* hybridization (FISH) (Fig. 1). FISH experiments with both probes labeled with biotin and detected as a red signals, showed that the number of sites of rDNA were constant among all the species under study. 5S rDNA *loci* were located in both arms of small chromosome pair in each species (Fig. 1). The hybridization sites of cloned 18S rDNA were associated with the secondary constriction of a large chromosome pair in each species, being a subtelocentric chromosome pair in *Agave tequilana* ‘Azul’ and a telocentric chromosome pair in *Agave cupreata* and *Agave angustifolia* ‘Lineño’ and ‘Cimarron’ (Fig. 1).

### Karyotype analysis

All the studied species were diploids with 2n = 2x = 60, confirmed by chromosome counting, considering the basic chromosome number x = 30 for the genus, and showed a bimodal karyotype with five pairs of large chromosomes and 25 pairs of small chromosomes. Karyotype analysis of *Agave* species is summarized in Table 1, and where it can be seen that all species showed different karyotypic formulae as well as a secondary constriction in one large chromosome pair; in *Agave tequilana* ‘Azul’ it was observed in pair 1, in *Agave cupreata* in pair 3, in *Agave angustifolia* ‘Lineño’ in pair 5 and in *Agave angustifolia* ‘Cimarron’ in pair 2.

**Table 1.** Karyotypes in *Agave* species (2n = 2x = 60).

**Taxa and origin**	**Collector and voucher information**	**Karyotype formula**	**Secondary constriction**
*Agave angustifolia* 'Cimarron' Tolimán, Jalisco State, México. 19°32'06"N, 103°53'44"W (DMS).	Rodríguez JM <br/> C	42m + 4sm + 6st + 8t^†^	2t
*Agave angustifolia* 'Lineño' Tolimán, Jalisco State, México. 19°32'06"N, 103°53'44"W (DMS).	Rodríguez JM <br/> L	48m + 2sm+ 2st + 8t^†^	2t
*Agave cupreata* Miraval, Guerrero State, México. 17°43'00"N, 99°45'00"W (DMS).	Trinidad RA<br/> 573	42m + 2sm + 8st + 8t^‡^	2t
*Agave tequilana* 'Azul' CIATEJ, Jalisco State, México. 20°41'39"N, 103°20'47"W (DMS).	Rodríguez JM<br/> A, C, D	42m + 12st + 6t^§^	2st

† = Palomino et al. unpublished data.

‡ = Karyotype published by Palomino et al. (2012).

§ = Karyotype published by Palomino et al. (2008).

**Figure 2.**
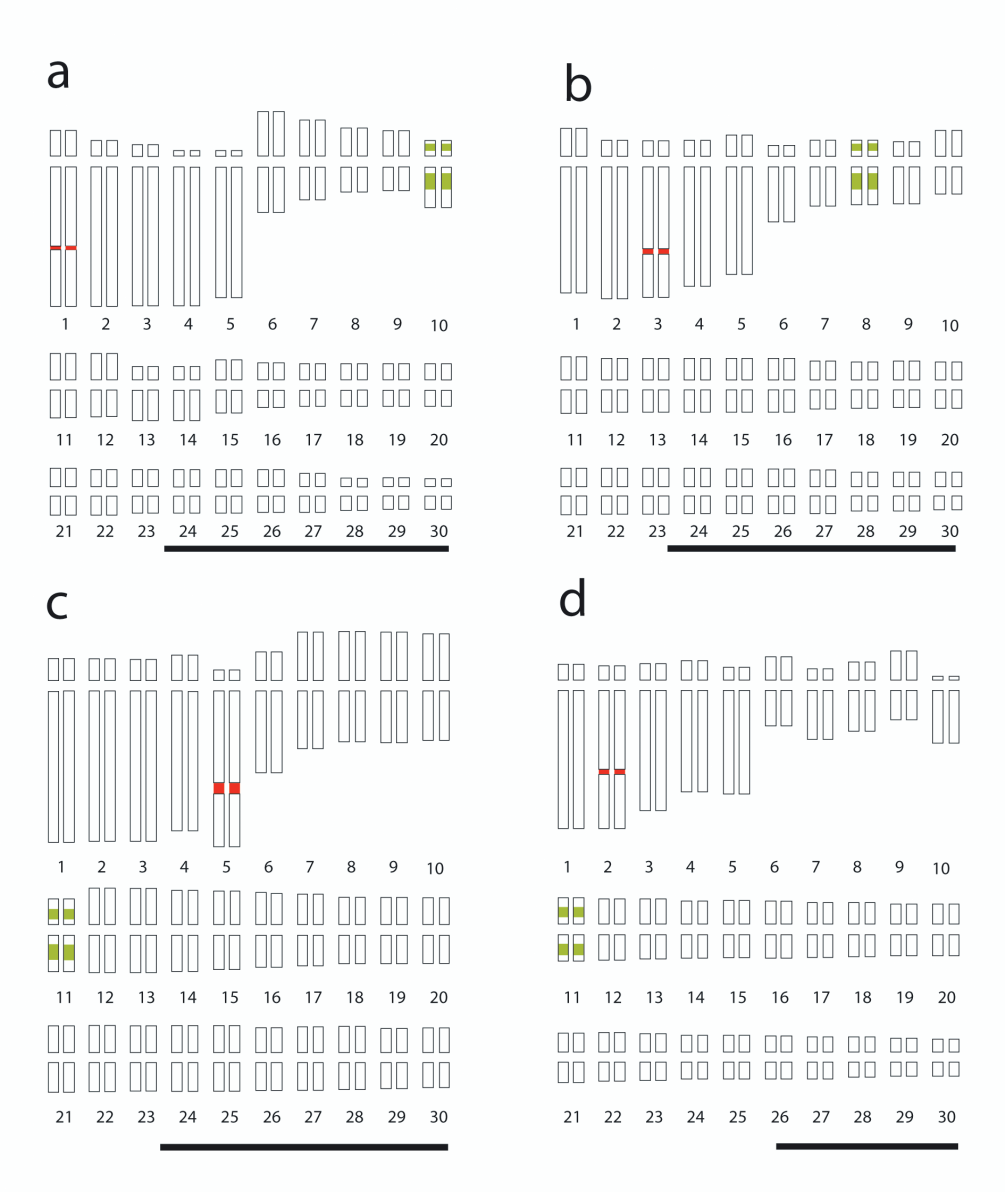
Idiograms of *Agave* karyotypes showing the 5S (green) and 18S (red) rDNA *loci*. **a** *Agave tequilana* ‘Azul’ **b** *Agave cupreata*; **c** *Agave angustifolia* ‘Lineño’ **d** *Agave angustifolia* ‘Cimarron’. Bars = 10 µm.

FISH data were integrated in idiograms, indicating the number and position of rDNA *loci* (Fig. 2). 5S rDNA *loci* always were located in a proximal region on both arms of a small chromosome in each species, whereas 18S rDNA *loci* always were located in the interstitial region of a large chromosome. Fig. 2a shows a hybridization signal of 18S rDNA in *Agave tequilana* ‘Azul’ on pair 1, while the 5S rDNA signals are on both arms of pair 10; in *Agave cupreata* (Fig. 2b), the hybridization signal of 18S rDNA is on pair 3, while the 5S rDNA signals are on both arms of pair 8; in *Agave angustifolia* ‘Lineño’ and ‘Cimarron’ (Fig. 2c-d), the hybridization signal of 18S rDNA is on pair 5 and 2, respectively, while the 5S rDNA signals are on both arms of pair 11 in both varieties.

## Discussion

Cytogenetic analysis showed the diploid chromosome number 2n = 60 in all species, which is in agreement with previous reports in the genus (Palomino et al. 2005, Palomino et al. 2008, Palomino et al. 2010). All species showed a bimodal karyotype with small and large chromosomes (n = S + L); this bimodal karyotype is shared among multiple genera in Asphodeloideae (Brandham and Doherty 1998, Adams et al. 2000, Vosa 2005) and Agavoideae (McKelvey and Sax 1933, Brandham 1969) and recently, McKain et al. (2012) demonstrated that the Agavoideae bimodal karyotype was originated by an allopolyploid event, where the progenitor species seems to be extinct. Despite maintaining the same karyotype in all species, it was also found different karyotype formulae. This inter- and intraspecific variation shown here has been reported in other species and varieties in the genus (Banerjee and Sharma 1988, Moreno-Salazar et al. 2007, Palomino et al. 2008), leading to the formation of different cytotypes. Moreno-Salazar et al. (2007) studied three wild populations of *Agave angustifolia* and found two different cytotypes; Palomino et al. (2008) analyzed eight varieties of *Agave tequilana* and reported the same number of cytotypes. The presence of different cytotypes in *Agave* genus could be originated by heterozygous chromosomal exchange (Moreno-Salazar et al. 2007, Palomino et al. 2008, Palomino et al. 2010), which can modify the structure of chromosomes and maintaining at the same time their diploid number (Lima-Cardoso et al. 2013).

FISH with rDNA probes showed that *loci* of 18S and 5S rDNA in *Agave* species were located in different chromosomes and on similar position in all species; this finding suggests that the chromosomes bearing the rDNA *loci* are homeologous and the difference in numerical assignment is due to chromosomal rearrangements as mentioned before. 18S rDNA *locus* always was located in the interstitial region on the large arm of a large chromosome and associated to the secondary constriction, whereas the 5S rDNA *loci* were located in a proximal region on both arms of a small chromosome in all species. These results differ from Robert et al. (2008) because they reported that *Agave* species have one *locus* of 5S rDNA by monoploid genome in some diploid and polyploid species in the genus, including *Agave tequilana* ‘Azul’ and *Agave angustifolia* ‘Letona’ (tetraploid) and *Agave angustifolia* ‘Chelem ki’ (hexaploid). The presence of 5S rDNA *loci* on both arms of a small chromosome in all species can be resulted from an unequal recombination or an event of transpositions; the latter have been reported previously in other monocots such as *Allium* Linnaeus, 1753 (Schubert and Wobus 1985), *Oryza* Linnaeus, 1753 (Shishido et al. 2000) and *Alstroemeria* Linnaeus, 1762 (Chacón et al. 2012). Recently, Khaliq et al. (2012), reported that Ty1-Copia retrotransposons are a major component of the *Agave tequilana* genome (approximately 32 %) and might played a vital role in the organization and evolution of it, which could explain the results reported here.

To the best of our knowledge, here we reported the number and location of rDNA *loci* in two species with no previous report, *Agave cupreata* and *Agave angustifolia* ‘Lineño’ and ‘Cimarron’ as well as a different *locus* of 5S rDNA in all species studied. Data of FISH analysis provides new information about physical mapping of rDNA in *Agave* and such identified sites can be useful as chromosome markers for chromosome identification in hybrids in breeding programs as well as in evolutionary studies.

## Conclusions

Despite the great diversity of the genus *Agave* which includes 166 species, the physical mapping of rDNA or other molecular markers are scarce, since just about five species have been described. The different karyotype formulae found in all species indicated the presence of cytotypes and data of FISH of rDNA allowed the physical mapping of *Agave cupreata* and two new varieties of *Agave angustifolia*. This work provides new information about the position and number of rDNA *loci* in *Agave* species through comparative karyotype analysis, however, further cytogenetic research must be conducted to understand the evolution of this genus and develop breeding programs to preserve its biodiversity.
